# Incorporating functional annotation with bilevel continuous shrinkage for polygenic risk prediction

**DOI:** 10.21203/rs.3.rs-2759690/v1

**Published:** 2023-04-12

**Authors:** Yongwen Zhuang, Na Yeon Kim, Lars G. Fritsche, Bhramar Mukherjee, Seunggeun Lee

**Affiliations:** University of Michigan–Ann Arbor; Seoul National University; University of Michigan–Ann Arbor; University of Michigan–Ann Arbor; Seoul National University

## Abstract

**Background:**

Genetic variants can contribute differently to trait heritability by their functional categories, and recent studies have shown that incorporating functional annotation can improve the predictive performance of polygenic risk scores (PRSs). In addition, when only a small proportion of variants are causal variants, PRS methods that employ a Bayesian framework with shrinkage can account for such sparsity. It is possible that the annotation group level effect is also sparse. However, the number of PRS methods that incorporate both annotation information and shrinkage on effect sizes is limited. We propose a PRS method, PRSbils, which utilizes the functional annotation information with a bilevel continuous shrinkage prior to accommodate the varying genetic architectures both on the variant-specific level and on the functional annotation level.

**Results:**

We conducted simulation studies and investigated the predictive performance in settings with different genetic architectures. Results indicated that when there was a relatively large variability of group-wise heritability contribution, the gain in prediction performance from the proposed method was on average 8.0% higher AUC compared to the benchmark method PRS-CS. The proposed method also yielded higher predictive performance compared to PRS-CS in settings with different overlapping patterns of annotation groups and obtained on average 6.4% higher AUC. We applied PRSbils to binary and quantitative traits in three real world data sources (the UK Biobank, the Michigan Genomics Initiative (MGI), and the Korean Genome and Epidemiology Study (KoGES)), and two sources of annotations: ANNOVAR, and pathway information from the Kyoto Encyclopedia of Genes and Genomes (KEGG), and demonstrated that the proposed method holds the potential for improving predictive performance by incorporating functional annotations.

**Conclusions:**

By utilizing a bilevel shrinkage framework, PRSbils enables the incorporation of both overlapping and non-overlapping annotations into PRS construction to improve the performance of genetic risk prediction. The software is available at https://github.com/styvon/PRSbils

## Background

Genetic data are important resources to improve the risk prediction for complex diseases(Khera *et al*., 2018). The genetic effects of variants across the genome can be summarized in the form of polygenic risk scores (PRS) that estimate individuals’ genetic liability. The wide availability of summary statistics from large-scale genome-wide association studies (GWAS) has facilitated the application of PRS(Pasaniuc and Price, 2017). Although PRS has shown great promise in the early identification and prediction of disease risks, its predictive performance remains limited for most diseases.

Genetic variants in different functional categories can have different shares of contribution to the heritability of complex traits, and recent studies have shown that the incorporation of functional annotation can improve the predictive performance of PRS(Hu *et al*., 2017; Marquez-Luna *et al*., 2020). In addition, for certain phenotypes where only a small proportion of variants are causal, PRS methods with Bayesian continuous shrinkage framework have been proposed to account for such sparsity(Ge *et al*., 2019). It is possible that the annotation group level effect is also sparse. Although existing PRS methods improve prediction accuracy through utilizing GWAS summary statistics and accounting for the potential sparsity of the genetic architectures, the number of PRS methods that incorporate 56 annotation information and apply continuous shrinkage on effect sizes is limited. For example, the sparsity assumption for the underlying genetic architectures is generally made either on a global level(Ge *et al*., 2019) or by partitioning variants into bins with similar sum of squared posterior mean effect sizes(Chun *et al*., 2020; Márquez-Luna *et al*., 2021), and there is a lack of study on addressing sparsity across the annotation groups.

In this paper, we propose a PRS method with bilevel continuous shrinkage prior to leverage the functional annotation information. This prior accommodates the varying genetic architectures both on the variant-specific level and on the functional annotation level, and the posterior update is conducted using a Gibbs sampler. PRSbils uses GWAS summary statistics instead of individual-level data and accounts for local LD patterns through an external LD reference panel. We conducted simulation studies and investigated the predictive performance in settings with different genetic architectures. We applied PRSbils to binary and quantitative traits in three real world data sources, and demonstrated that the proposed method holds the potential for improving predictive performance by incorporating functional annotations.

## Methods

### Overview of Bayesian continuous shrinkage regression model

Denote *y* as an *N*-vector of standardized phenotype, *G* as an *N* × *M* matrix of standardized genotypes, *ϵ* as an *N*-vector of random noise, and *β* as an *M* vector of genetic effect sizes. Then, a regression model of genotypes and phenotypes can be expressed as

y=Gβ+ϵ

where *ϵ* ~ *MVN*(0, *σ*^2^*I*_*N*_), *p*(*σ*^2^) ∝ *σ*^−2^, i.e., *y*|*G*, *β*, *σ*^2^ follows a multivariate Normal distribution with mean *Gβ* and covariance matrix *σ*^2^*I*_*N*_.

For high dimensional genetic data, the number of genetic variants *M* is much larger than the number of individuals *N*, and it is often assumed that the genetic effect vector *β* is sparse, meaning that only a small amount of the variants are associated with the outcome phenotype. Under this sparsity assumption, the prior distribution of *β* can be chosen to be either a discrete or a continuous mixture of Normal distributions. The discrete mixture type of prior is also known as the spike-and-slab prior(George and McCulloch, 1993), and is a combination of a point mass at 0 and a density for the non-zero part.

The continuous mixture type of prior assigns *β* with a continuous distribution centered at 0. One commonly used set of priors is the global-local shrinkage priors(Polson and Scott, 2010), which utilizes both a global shrinkage parameter *τ*^2^ and local marker-specific parameters $\lambda_{1}^{2},\ldots ,\lambda_{M}^{2}$ to model the prior distribution of *β*. Specifically:

$$\beta |\sigma^{2},\tau^{2},\lambda_{1}^{2},\ldots ,\lambda_{M}^{2}\sim MVN\left(0,\frac{\sigma^{2}}{N}\tau^{2}V_{\lambda }\right)$$

$$\lambda_{j}^{2}\sim \pi_{1}\left(\lambda_{j}^{2}\right)d\lambda_{j}^{2},j\in \{1,\ldots ,M\}$$

$$\tau^{2}\sim \pi_{2}\left(\tau^{2}\right)d\tau^{2}$$

where $V_{\lambda }\equiv diag\left\{\lambda_{1}^{2},\ldots ,\lambda_{M}^{2}\right\}$ is an M × M diagonal matrix, and *π*_1_ and *π*_2_ are absolutely continuous functions and have a wide range of choices. For example, the model becomes Lasso when $\lambda_{j}^{2}$ follows the standard exponential distribution; a horseshoe prior is constructed when both *τ* and *λ*_*j*_ follow a standard half-Cauchy distribution.

The above-mentioned Bayesian model can be generalized to the bilevel global-local shrinkage models to account for additional group information(Xu *et al*., 2017). We consider the situation where each variant belongs to one of *K* mutually-exclusive annotation groups, i.e., *A*_*j*_ = *k* if variant *j* belongs to annotation group *k*. Then the Bayesian bilevel global-local shrinkage regression model can be expressed as:

$$\beta |\sigma^{2},\delta_{A_{1}}^{2},\ldots ,\delta_{A_{M}}^{2},\lambda_{1}^{2},\ldots ,\lambda_{M}^{2}\sim MVN\left(0,\frac{\sigma^{2}}{N}V_{\delta }V_{\lambda }\right)$$

$$\lambda_{j}^{2}\sim \pi_{1}\left(\lambda_{j}^{2}\right)d\lambda_{j}^{2}$$

$$\delta_{k}^{2}\sim \pi_{2}\left(\delta_{k}^{2}\right)d\delta_{k}^{2},k\in \{1,\ldots ,K\}$$

where $V_{\delta }\equiv diag\left\{\delta_{A_{1}}^{2},\ldots ,\delta_{A_{M}}^{2}\right\}$, with $\delta_{A_{j}}^{2}$ being the group-level shrinkage parameter when the corresponding group for the *j*th variant is *A*_*j*_.

### PRSbils

Based on the Bayesian bilevel global-local shrinkage regression model, we incorporate functional annotation as the group-level information and assume a standard half-Cauchy prior *C*^+^(0,1) for each group-level shrinkage parameter *δ*_*k*_ and each local shrinkage parameter *λ*_*j*_. The Bayesian regression model can then be specified as:

$$y|G,\beta ,\sigma^{2}\sim MVN\left(G\beta ,\sigma^{2}I_{N}\right)$$

$$\beta |\sigma^{2},\delta_{1}^{2},\ldots ,\delta_{K}^{2},\lambda_{1}^{2},\ldots ,\lambda_{M}^{2}\sim MVN\left(0,\frac{\sigma^{2}}{N}V_{\delta }V_{\lambda }\right)$$

$$\sigma^{2}\sim \sigma^{-2}d\sigma^{2}$$

$$\delta_{k}\sim C^{+}(0,1)$$

$$\lambda_{j}\sim C^{+}(0,1)$$

The posterior distribution of *β* and *σ*^2^ can then be derived:

$$\beta |\cdot \sim MVN\left(B^{-1}G^{T}y,\sigma^{2}B^{-1}\right)$$

$$\sigma^{2}|\cdot \sim IG(\frac{n+p-1}{2},\frac{1}{2}\left[{\left(y-G\beta \right)}^{T}(y-G\beta )+\beta^{T}{\left(V_{\delta }V_{\lambda }\right)}^{-1}\beta \right)$$

where *B* = *G*^*T*^*G* + (*V*_*δ*_*V*_*λ*_)^−1^.

When the LD matrix *D* and the summary-level estimation of genetic effect size $\hat{\beta }$ are available, we can obtain an approximation for the posterior distribution with $\hat{\beta }=G^{T}y/N$ and *D* = *G*^*T*^*G*/*N*:

$$\beta |\cdot \sim MVN\left(\frac{N}{\sigma^{2}}\text{Σ}\hat{\beta },\text{Σ}\right)$$

$$\sigma^{2}|\cdot \sim IG\left(\frac{1}{2}(N+M),\frac{N}{2}\left\{1-2\beta^{T}\hat{\beta }+\beta^{T}\left[D+{\left(V_{\delta }V_{\lambda }\right)}^{-1}\right]\beta \right\}\right)$$

where $\text{Σ}=\frac{\sigma^{2}}{N}{\left[D+{\left(V_{\delta }V_{\lambda }\right)}^{-1}\right]}^{-1}.$.

To derive the posterior distributions for shrinkage parameters *δ* and *λ*, we note that the standard half-Cauchy distribution can be decomposed into a scale mixture of inverse Gamma distributions(Makalic and Schmidt, 2015). Let *x*, *α* be random variables satisfying

$$x^{2}|a\sim IG\left(\frac{1}{2},a\right),\;a\sim IG\left(\frac{1}{2},1\right)$$

where *IG*(*α*, *β*) is the inverse Gamma distribution with shape parameter *α* and scale parameter *β*, then *x* ~ *C*^+^(0,1).

#### PRSbils with non-overlapping annotation assignment.

We first consider the situation where each variant has only one annotation. Using the scale mixture representation of the standard half-Cauchy distribution, we obtain an alternative representation of the priors for *δ* and *λ*:

$$\delta_{k}|t_{k}\sim IG\left(\frac{1}{2},\frac{1}{t_{k}}\right),t_{k}\sim IG\left(\frac{1}{2},1\right),k\in \{1,\ldots ,K\}$$

$$\lambda_{j}|c_{j}\sim IG\left(\frac{1}{2},\frac{1}{c_{j}}\right),c_{j}\sim IG\left(\frac{1}{2},1\right),j\in \{1,\ldots ,M\}$$

The posteriors for *δ* and hyper-parameter *t* can be expressed as

$$\delta_{k}^{2}|\cdot \sim IG\left(\frac{M_{k}+1}{2},\frac{\sum_{j\in \left\{j:A_{j}=k\right\}}\frac{N\beta_{j}^{2}}{\lambda_{j}^{2}}}{2\sigma^{2}}+t_{k}\right)$$

$$t_{k}|\delta_{k}^{2}\sim IG\left(1,\delta_{k}^{-2}+1\right)$$

where *M*_*k*_ denotes the number of variants within group *k*, *k* = 1, …, *K*.

We obtain the posteriors for *λ* and *c* in a similar fashion:

$$\lambda_{j}^{2}|\beta_{j},\sigma^{2},\delta_{A_{j}}^{2},c_{j}\sim IG\left(1,\frac{N\beta_{j}^{2}}{2\sigma^{2}}+c_{j}\right)$$

$$c_{j}|\lambda_{j}^{2},\delta_{A_{j}}^{2}\sim IG\left(1,\lambda_{j}^{-2}+1\right)$$

Since all the conditional distributions for the parameters are known, posterior samples of *β* can be obtained by a Gibbs sampler. After the posterior *β* values (denoted as $\tilde{\beta }$) are achieved, we construct the final PRS score by combing the group-wise scores across all annotations

$$PRS=\sum_{k=1}^{K}\alpha_{k}PRS_{k}$$

where $PRS_{k}=\sum_{\left\{j:A_{j}=k\right\}}G_{j}{\tilde{\beta }}_{j}$ is the group-wise score, and *α*_*k*_ is the group-specific weight for group *k*. We obtain the estimates for *α*_1_, …, *α*_*k*_ through 10-fold cross-validation using a separate validation data, which includes individual-level genotype and phenotype data. We use this group-wise combination approach based on the observation that signal-enriched annotations are more informative for prediction, and weighting each partition differently can further improve PRS performance(Chun *et al*., 2020).

We also investigated a hybrid method that is a combination of PRSbils and conventional PRS methods. In this case, we construct the PRS score by

$$PRS=\sum_{k=1}^{K}\alpha_{k}PRS_{k}+\gamma \sum_{j=1}^{M}G_{j}{\tilde{\beta }}_{j}^{\prime }$$

where ${\tilde{\beta }}_{j}^{\prime }$ denotes the genetic effect size generated from conventional PRS methods.

#### PRSbils with overlapping annotation assignment

Under the situation where a variant belongs to multiple annotation groups, for example, one variant can be involved in multiple pathways, we account for such a variant separately in each of the annotation groups it belongs to. The assumption underlying this is that variants with more annotations have potentially larger contribution to the heritability and therefore will be shrunk less and have larger posterior effect sizes in general(Chen *et al*., 2020). The total number of variants in the overlapping setting will become $M^{\prime }=\sum_{k=1}^{K}\sum_{j=1}^{M}I\left(k\in A_{j}\right)$, and the local shrinkage parameters will be assigned to each of the *M*′ variants:

$$\lambda_{j,A_{j}}^{2}|\beta_{j,A_{j}},\sigma^{2},\delta_{A_{j}}^{2},c_{j,A_{j}}\sim IG\left(1,\frac{N\beta_{j,A_{j}}^{2}}{2\sigma^{2}}+c_{j,A_{j}}\right)$$

$$c_{j,A_{j}}|\lambda_{j,A_{j}}^{2},\delta_{A_{j}}^{2}\sim IG\left(1,\lambda_{j,A_{j}}^{-2}+1\right)$$

The group-wise score can be calculated by $PRS_{k}=\sum_{\left\{j:A_{j}=k\right\}}G_{j}{\tilde{\beta }}_{j,A_{j}}.$

### PRS-CS

PRS-CS is a PRS method which infers the posterior genetic effect size *β* using summary-level $\hat{\beta }$ from existing GWAS studies as well as LD information from a reference panel. It is based on the Bayesian continuous shrinkage regression model without group information and obtains posterior samples using a Gibbs sampler. A general gamma-gamma distribution is assigned to the local shrinkage parameters $\lambda_{j}^{2}$:

$$\lambda_{j}|c_{j}\sim G\left(a_{0},c_{j}\right),c_{j}\sim G\left(b_{0},1\right)$$

with *G*(·,·) representing a Gamma distribution with shape and scale parameters, and *α*_0_ and *b*_0_ being pre-specified constants. When *α*_0_ = 0.5 and *b*_0_ = 1, it is equivalent to the scale mixture representation of the standard half-Cauchy distribution.

When a prior guess of the global shrinkage parameter *τ* is not available, PRS-CS either uses a grid search for the best performing value in an additional validation set (PRS-CS), or assigns a standard half-Cauchy prior on *τ* in the fully Bayesian model (PRS-CS-auto). For the posterior sampling part, PRSbils is an extension of the PRS-CS-auto approach to differentiate the shrinkage across different groups. When the number of groups *K* = 1, our approach is equivalent to PRS-CS-auto with hyper-parameters *α*_0_ = 0.5 and *b*_0_ = 1.

### LDpred-funct

LDpred-funct incorporates functional annotation as priors for the genetic effects using the baseline-LD model which includes non-overlapping annotations(Márquez-Luna *et al*., 2021). It assumes a prior distribution $\beta_{j}\sim N\left(0,c\sigma_{j}^{2}\right)$ for the normalized genetic effects, where $\sigma_{j}^{2}$ represents per-SNP heritability obtained from stratified LD score regression(Bycroft *et al*., 2018) and *c* is a normalizing constant. The posterior mean of *β* is

$$E[\beta |\cdot ]=W^{-1}N\hat{\beta }$$

Where $W^{-1}={\left[ND+\frac{1}{c}diag\left(\frac{1}{\sigma_{1}^{2}},\ldots ,\frac{1}{\sigma_{M}^{2}}\right)\right]}^{-1}$. The SNPs are then ranked by the absolute posterior mean effect sizes and partitioned into *L* bins with approximately the same sum of squared posterior mean effect sizes. The PRS is generated by

$$PRS=\sum_{l=1}^{L}\alpha_{l}PRS(l)$$

### where the weights are determined via 10-fold cross-validation.

To make LDpred-funct applicable to our study which uses different annotations from the baseline LD model, we used stratified LD score regression to obtain the per-SNP heritability under the functional annotations being used, obtained the posterior mean of *β*, and get the PRS with the number of bins *L* fixed at 40.

### Biobank data overview

UK Biobank is a large-scale database with biomedical information from UK participants recruited from 2006 to 2010(Bycroft *et al*., 2018). The genetic data from UK Biobank consists of over 90 million genetic variants imputed from the Haplotype Reference Consortium (HRC)(McCarthy *et al*., 2016) among 488,377 individuals. Data from the Michigan Genomics Initiative (MGI)(Fritsche *et al*., 2018) and the Korean Genome and Epidemiology Study (KoGES) data(Kim *et al*., 2017) were also analyzed in our study. We used Data Freeze 3 of the MGI data, which includes 56,984 genotyped participants at the University of Michigan with over 32 million genome-wide variants imputed from the HRC. The KoGES data includes a total of 72,298 Korean individuals, with over 8 million genetic variants imputed from 1,000 Genome project phase 3 + Korean reference genome (397 samples) and with minor allele frequency (MAF) > 0.01, HWE p-value > 1 × 10^−6^, variant call rate > 95%.

For all genetic data in the UK Biobank, MGI and KoGES, NCBI Build 37/UCSC hg19 was used for genomic coordinates. We further restricted our analysis to HapMap3 SNPs with minimum MAF > 0.01, HWE p-value > 1 × 10^−6^, variant call rate > 95%, individual missing rate < 1%, and LD-pruning R2<0.99. LD information from 503 European samples in the 1000 Genomes Project (1KG)(1000 Genomes Project Consortium *et al*., 2010) was used as an external reference panel for the UK Biobank and MGI data, and 1KG East Asian reference panel was used for the KoGES data.

### Simulation studies

We conducted simulation studies to compare the performance of PRSbils to PRS-CS. We also evaluated a hybrid method of PRSbils with PRS-CS, in which the scores from PRS-CS was combined with the one from the proposed method. We also compared the predictive performance with LDpred-funct for non-overlapping annotation groups. A total of *M* = 125,000 SNPs were sampled from the UK Biobank data with above-mentioned quality control filters, with 1KG as LD reference panel. The sampled variants were then assigned to *K* different annotation groups, which explain *q*_1_, …, *q*_*K*_ % of the total heritability *h*^2^ respectively. For each annotation group *k*, the proportion of causal variants is denoted as *p*_*k*_. Genetic effect sizes were generated from a mixture of point-Normal models specified as:

$$\beta_{j}\sim \{\begin{array}{l} N\left(0,\frac{q_{A_{j}}h^{2}}{p_{A_{j}}M}\right),\text{ with probability }p_{A_{j}}\\ 0,\text{ with probability }1-p_{A_{j}} \end{array}$$

We investigated five simulation settings with different *K*, *p*_*k*_ and *q*_*k*_ with non-overlapping annotation groups, i.e., each variant is mapped to one and only one annotation (Table 1). For settings 1–4, we fixed the number of annotation groups to 4 (*K* = 4), the proportion of causal variants in each group to 0.5%, 1%, 1.5%, 2% respectively, and vary the proportion of the total heritability explained by each group from a relatively sparse scenario (*q* = (0,0,10%, 90%)) to a more balanced scenario (*q* = (25%, 25%, 25%, 25%)). For setting 5, we changed the number of annotation groups to *K* = 10, and considered a situation with more group-wise sparsity where only two of the groups contribute to the total heritability.

In addition, we simulated two settings (settings 6 and 7 in Table 1) with different overlapping patterns (**Supplementary Figure 1**). For both settings, we used four annotation groups contributing 0,0,10%,90% to the total heritability. Overlapping pattern I was used in setting 6, where the intersection over union metric (IOU) was higher among the annotation groups with low heritability contribution. For setting 7, overlapping pattern II was used, where IOU was higher among the annotation groups with high heritability contribution.

We then simulated the phenotypes using the sum of all SNPs weighted by their corresponding genetic effect sizes, together with a Normal random error term to fix the heritability at *h*^2^ = 0.7.

To obtain the summary statistics, we performed GWAS to calculate the marginal genetic effect size estimates $\hat{\beta }$ using SAIGE^20^ version 0.44.3, which is a computationally efficient method that controls for case-control imbalance as well as potential sample relatedness, among *N*_*sumstαt*_ = 50,000 simulated individuals. The summary statistics were used as input for PRSbils and PRS-CS.

The prediction performance was evaluated for both methods on a separate test set consisting of *N*_*test*_ = 24,000 simulated individuals. AUC and *R*^2^ were used to measure the prediction accuracy. To obtain AUC, we binarize the phenotypes assuming those with top 10% highest phenotype values as the true at-risk population.

### Biobank data analysis

We analyzed both binary and quantitative traits for three data sources, i.e., the UK Biobank data, the MGI data, and the KoGES data. For the UK Biobank and the MGI data, we studied type II diabetes as a binary trait, and BMI and LDL as quantitative traits. For the KoGES data, we assessed the results for type II diabetes. The binary type II diabetes trait for genotyped individuals were defined by the PheWAS codes(Bastarache, 2021) aggregated from ICD codes in the electronic health records for the UK Biobank data and the MGI data, while for the KoGES data it is identified from questionnaire-based interviews. The quantitative traits were obtained as a physical measure in the target data at the initial assessment visit of the participants. For each trait in the analysis, we used a common set of SNPs from the summary statistics, the 1000G reference panel, and the test set. The total number of SNPs used was 1,093,109 for type II diabetes, 986,885 for BMI, and 926,775 for LDL.

The summary statistics used in the analysis were from existing study results. For type II diabetes, we used the result from a GWAS analysis of 407,701 white British UK Biobank participants using SAIGE(Zhou *et al*., 2018) when analyzing UK Biobank and MGI data, while summary statistics from Biobank Japan was used for the KoGES data. For BMI, we used the GWAS results from GIANT Consortium with 332,153 participants with European ancestry(Turcot *et al*., 2018). For LDL, GWAS results from the GLGC Consortium with 188,578 participants with European ancestry(Willer *et al*., 2013) were used.

To avoid the overlapping of samples between the test samples and the samples in the UK Biobank summary statistics for type II diabetes, we applied the PRS methods to a sample of 7,528 white individuals with non-British origin in the UK Biobank. For quantitative traits, since the summary statistics does not overlap with the UK Biobank population, we applied the methods to a test set consisting of around 80,000 white British individuals in the UK Biobank. Two types of group information were used for PRSbils. The first was 186 pathway annotations from the Kyoto Encyclopedia of Genes and Genomes (KEGG), which includes the networks for metabolism, genetic information processing, environmental information processing, cellular processes, organismal systems, human diseases and drug development(Kanehisa and Goto, 2000). Variants were first mapped to Ensembl genes by position, and then from genes to KEGG pathways. The mapping of genetic variants to KEGG annotations is not unique, which means each variant can have multiple KEGG annotations. The second one was six non-overlapping Refseq gene-based functional annotations (i.e. exonic/splicing; ncRNA; UTR5/ UTR3; intronic; upstream/ downstream; intergenic) obtained using ANNOVAR(Wang *et al*., 2010). For variants without any available annotations, PRSbils assigned them to a separate group for no annotations.

For each trait, we adopted a 10-fold cross-validation approach where the *α* parameters were estimated using a random sample of 9/10 of the test set, and the performance was validated on the rest 1/10 of the samples in terms of *AUC* and *R*^2^. For the binary trait, we used Efron’s pseudo *R*^2^(Efron, 1978) instead of the ordinary least square *R*^2^. For the continuous traits BMI and LDL, we calculated the AUC by binarizing them with thresholds of 25 (threshold for overweight) and 4.1 mmol/L (or 160mg/dL, threshold for high LDL) respectively.

## Results

### Simulation study results

We evaluated prediction performance of PRSbils, PRS-CS and the hybrid method in seven simulation settings (Table 1). For the settings with non-overlapping annotation groups (Settings 1–5), we also evaluated the performance of LDpred-funct. Since the estimated per-SNP heritability might not be stable due to the relatively small sample size, we used the true stratified heritability values for LDpred-funct.

**Figure 1.**
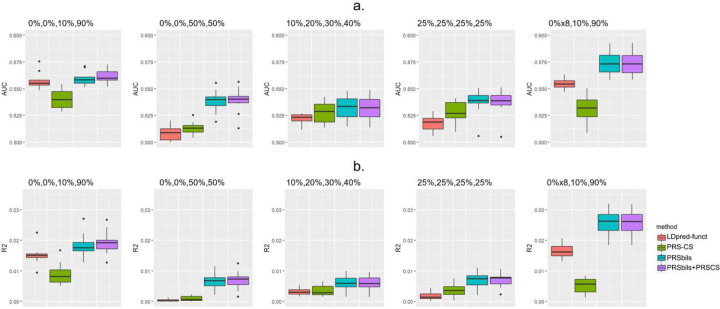
Comparison of prediction performance in simulation studies with non-overlapping annotation groups (Settings 1–5), measured by AUC (a) and *R*^2^ (b). A total of *M* = 125,000 SNPs were sampled from the UK Biobank data with 1KG as LD reference panel. Genetic effects were generated using a mixture of point-Normal models with total heritability fixed at 0.7. GWAS results from *N*_*sumstαt*_ = 50,000 simulated individuals were used as summary statistics. Prediction accuracy was evaluated in a test sample of *N*_*test*_ = 24,000 simulated individuals with 10-fold cross-validation.

Simulation Settings 1–4 consider a fixed number of total annotation groups *K* = 4. Figure 1 shows that when there is a relatively large variability of group-wise heritability contribution, the gain in prediction performance from PRSbils is the largest compared to PRS-CS. For example, in setting 1, where each annotation group contribute 0%, 0%, 10% and 90% of the total heritability, PRSbils yielded an average AUC of 0.559 (95%CI: [0.546,0.572]), the hybrid PRSbils+PRS-CS method yielded an average AUC of 0.561 (95%CI: [0.548,0.575]), compared to an average AUC of 0.527 (95%CI: [0.500,0.555]) from PRS-CS and 0.540 (95%CI: [0.523,0.558]) from LDpred-funct. Similar patterns of AUC were shown in other settings. The improved average performance over the benchmark methods can be explained by the group-wise shrinkage parameter allowing for shrinking the group with high heritability contribution differently than other annotation groups, instead of making a uniform shrinkage at the global level. When the difference in group-wise heritability contribution was small, such as in Setting 4 where all groups contribute equally to the total heritability, the prediction performance of PRSbils was similar to PRS-CS. This is expected as the group-wise shrinkage parameters from PRSbils would behave similarly as the global shrinkage parameter in PRS-CS.

In Setting 1 and Setting 5, two groups contribute 10% and 90% to the total heritability, but the total number of annotation groups differ (*K* = 4 in Setting 1 and *K* = 10 in Setting 5). PRSbils yields similar performance in these two settings, but has a slightly larger variability in Setting 5, which yielded with an average AUC of 0.574 (95%CI: [0.552,0.595]) (Figure 1). This is likely because the proposed method only shrinks groups with no heritability contribution to a small value but not exactly to zero, and therefore the large proportion of no heritability annotation groups, the noisier the PRS value will be, making the performance to fluctuate more.

**Figure 2.**
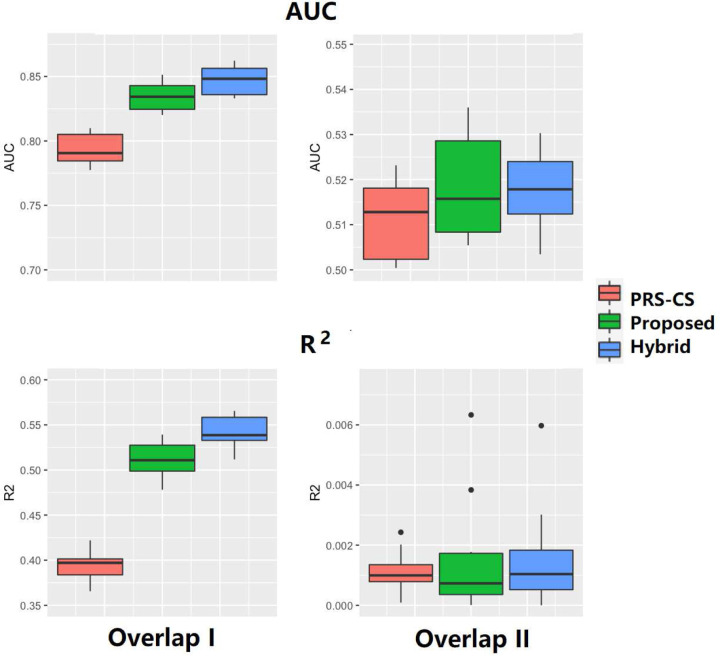
Comparison of prediction performance in simulation studies with overlapping annotation groups (Settings 6–7), measured by AUC (a) and *R*^2^ (b). Left: Setting 6 with overlapping pattern I, in which IOU is higher among the annotation groups with low heritability contribution. Right: Setting 7 with overlapping pattern II where IOU is higher among the annotation groups with high heritability contribution.

In Settings 6 and 7, we investigated the influence of using different overlapping patterns of annotation groups on the performance (Figure 2). PRSbils yielded higher predictive performance compared to the benchmark method in both settings, with an average 8.4% gain in AUC for Setting 6 and 0.8% for Setting 7. An explanation for the difference in the performance gain is that the high IOU between annotation group 3 and group 4 in Setting 7 resulted in a higher correlation in the group-wise shrinkage parameters, which reduced PRSbils’s ability to differentiate between these groups with different heritability contribution. These results suggest that under the overlapping annotation scenario, choosing an annotation mapping which better separates the potential heritability-contributing sets may improve the prediction accuracy.

### Biobank data analysis

**Figure 3.**
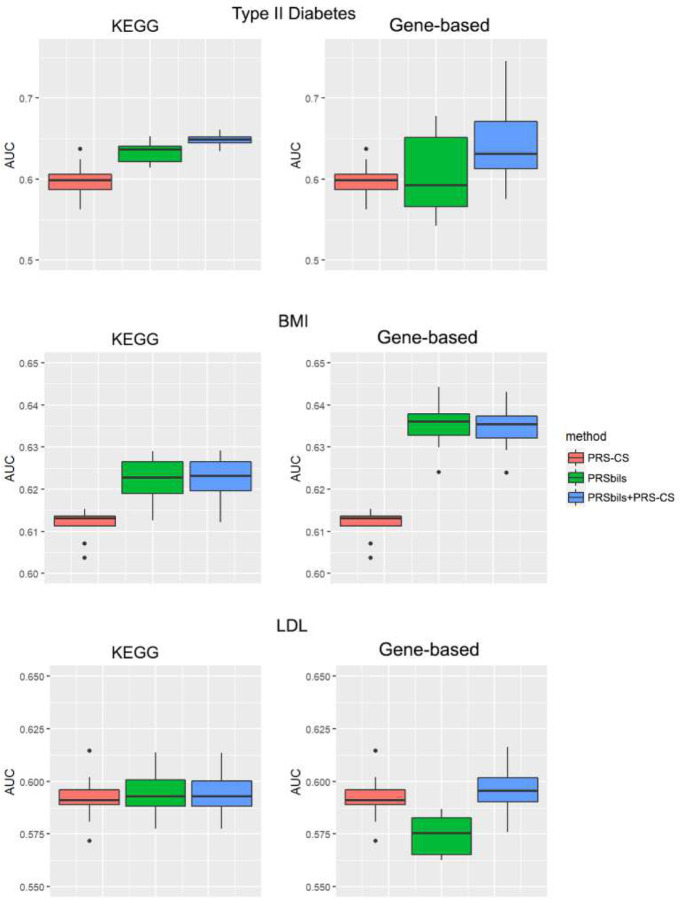
Evaluation of AUC for UK Biobank analysis results. Left panel: KEGG functional annotations were used for the analysis of the proposed; Right panel: Refseq gene-based functional annotations from ANNOVAR were used for the analysis of PRSbils. From top to bottom: type II diabetes, BMI, LDL. For type II diabetes, summary statistics were obtained result from a GWAS analysis of 407,701 white British UK Biobank participants. For BMI, we used the GWAS results from GIANT Consortium with 332,153 participants with European ancestry. For LDL, GWAS results from the GLGC Consortium with 188,578 participants with European ancestry were used.

**Figure 4.**
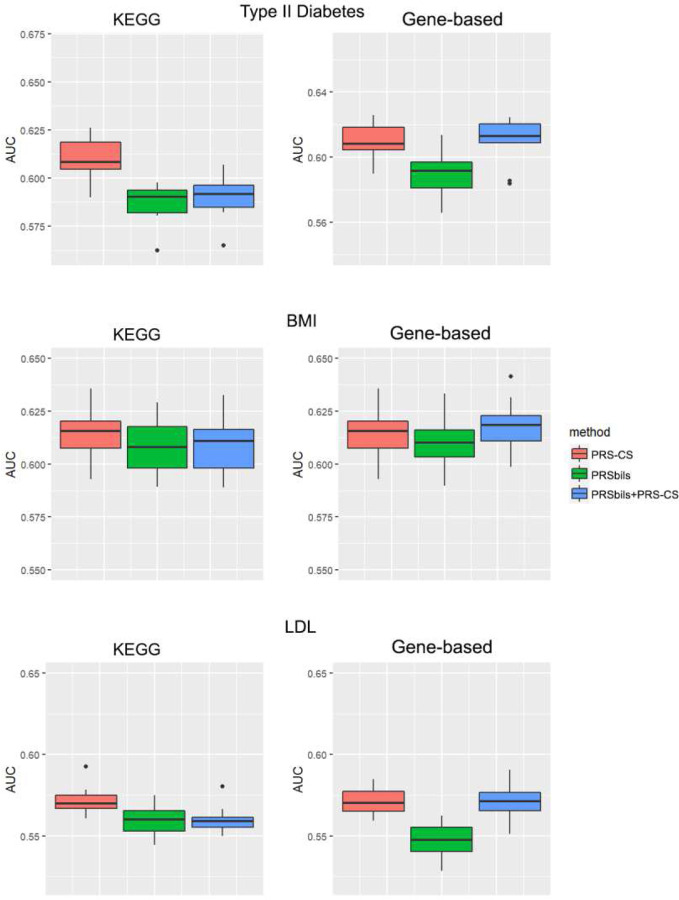
Evaluation of AUC for the MGI data. Left panel: KEGG functional annotations were used for the analysis of the proposed; Right panel: Refseq gene-based functional annotations from ANNOVAR were used for the analysis of PRSbils. a: type II diabetes; b: BMI; c: LDL. For type II diabetes, summary statistics were obtained result from a GWAS analysis of 407,701 white British UK Biobank participants. For BMI, we used the GWAS results from GIANT Consortium with 332,153 participants with European ancestry. For LDL, GWAS results from the GLGC Consortium with 188,578 participants with European ancestry were used.

We used the summary statistics as the training data to obtain the posterior genetic effect estimates, and evaluated the performance of both the proposed and existing PRS methods using the UK Biobank data (Figure 3, **Supplementary Figure 2**), the MGI data (Figure 4, **Supplementary Figure 3**) and the KoGES data(**Supplementary Figure 4**) as the test data. Each box plot contains the 10 results from the 10-fold cross validation using the test data.

For the UK Biobank data, PRSbils with KEGG annotation outperformed PRS-CS for both type II diabetes and BMI. PRSbils yielded a 9.9% improvement in AUC over PRS-CS for type II diabetes on average, and a 1.5% average improvement for BMI. When gene-based annotations derived from ANNOVAR were used, *AUC* from PRSbils was on average 3.8% higher than PRS-CS for BMI, yet the predictive performance for type II diabetes was similar among the methods. For only one scenario with LDL trait using gene-based annotation, PRSbils yielded lower prediction performance than PRS-CS. We also note that the prediction performance varies across different populations for different annotation information. For the MGI and KoGES data, PRSbils did not yield better prediction performance than the benchmark method for the traits analyzed. One potential factor that can lead to this difference in performance is the cohort difference: The UKB is population-based and consists of UK participants mainly of European ancestry, while the MGI is patient-based, and the KoGES is an East Asian cohort. Such a difference may lead to different distributions of risk-contributing annotation groups and consequently affect the predictive result. In contrast, the hybrid approach of PRS-CS and PRSbils performed robustly, showed high performance in most of the analysis.

It is likely that different annotation types vary in their contribution to the total heritability for diseases of interest, and as indicated by the simulation studies, such a difference affects the performance of the group-wise shrinkage parameter from the proposed method compared to the global shrinkage parameter in PRS-CS. We investigated the overall shrinkage of the two methods and presented the results in **Supplementary Figure 5**.

We also performed an additional analysis to compare the performance with LDpred-funct(Márquez-Luna *et al*., 2021). Due to the lack of availability of the functional enrichment files for annotations other than those in the baselineLD model as required by LDpred-funct, we only compared the performance using the baselineLD model annotations, with type II diabetes as the phenotype. The predictive performance measured in AUC were similar across all the methods compared (**Supplementary Figure 6**) for the annotations in the baselineLD model, while LDpred-funct was not able to analyze annotations from KEGG.

### Computation time

Computation time was evaluated for the UK Biobank data analysis for Type II diabetes with KEGG annotation. PRSbils yielded a computation time of 14.0 CPU hours, compared to 12.9 CPU hours for PRS-CS. The slight increment of computation time for PRSbils is largely due to the additional computation on variants with overlapping annotation groups. All evaluations were computed on an Intel(R) Xeon(R) Gold 6242R CPU.

## Discussion

PRSbils can be applied to both non-overlapping and overlapping annotations. When the annotation categories overlap with each other (i.e., one SNP can belong to multiple annotation categories), the posterior effect size is calculated and incorporated into the PRS separately for each category a variant belongs to. The underlying assumption for this framework is that SNPs belonging to more annotation categories are prioritized for genetic risk calculation as they are more likely to be causal. It is similar to the idea of penalizing the

SNPs with multiple annotations less than those with only one annotation category in a penalized regression framework(Chen *et al*., 2020). As has been illustrated in the simulation studies, sparsity of the underlying heritability enrichment from each annotation group is a key factor for the predictive performance of PRSbils. When multiple groups are included in an annotation categorization, PRSbils is expected to yield a larger performance improvement than the methods not utilizing annotation group information if only a few groups contribute a relatively large proportion of the heritability. This is because the group shrinkage parameter from PRSbils is able to differentiate the degree of shrinkage across the annotation groups, instead of putting a uniform global shrinkage for all variants. Recent studies have shown that such group sparsity patterns are present in human traits. For example, pathway analysis of GWAS suggested that genetically associated variants are enriched in specific genes or pathways for traits such as diabetes, schizophrenia, and Alzheimer’s disease.(Mei *et al*., 2018; Pardiñas *et al*., 2018; Jansen *et al*., 2019) We expect that identifying and applying these group-sparse annotations on a disease by disease basis would help further improve predictive performance.

In addition, the application of the proposed method using the KEGG annotations explores the pathway-level knowledge of polygenic risk for complex diseases, and provides an alternative way to stratify genetic liability in addition to the commonly used functional annotation, which adds to existing literature’s ongoing investigation into the use of pathway PRS as a more informative way for patient stratification and treatment response prediction(Choi *et al*., 2021). The average shrinkage for each KEGG annotation group can provide biological or clinical interpretations such as how different pathways weigh in terms of their relative importance for disease risk prediction. We illustrate this with the group-wise average shrinkage results from the UKB analysis (**Supplementary Figure 7**).

The shrinkage parameters *δ*_*k*_ in PRSbils control the degree of shrinkage across annotation categories, and are automatically learned from the summary statistics in this study. An alternative way to specify the values for *δ*_*k*_ is to fix them using prior knowledge about the annotation-level sparseness of the genetic architecture. If the sample size of the training set to train *α* is small, we expect the latter approach to yield higher predictive performance, because the current fully Bayesian approach would generate less stable estimates for the shrinkage parameters under this situation. Indeed, with additional simulations we confirmed that the performance of the current PRSbils approach would have a higher variance when the sample size for the training set was small (**Supplementary Figure 8**).

We also explored the influence of annotation misclassification on prediction performance through additional simulation studies. Genotype and phenotype data were generated using the same settings as in Settings 1–5 (Table 1) with “true” corresponding annotation group assignment. Then, the variants were assigned a random “observed” annotation group with equal probability to train and test for prediction performance. The results showed that misclassification of annotation groups have negative impact on the predictive performance of PRSbils, especially when there is larger group sparsity in each annotation group’s contribution to heritability (**Supplementary Figure 9**). Thus, to achieve good performance, it is important to ensure that the group annotations well depicts the underlying heritability structure.

We note several points that can be further improved in future studies for PRSbils. Firstly, from the predictive performance of PRSbils evaluated in UK Biobank, MGI, and KoGES data, we noted that the results were not consistent across different data sources, which indicates the influence of cohort difference over predictive performance when annotation information is incorporated. It is thus critical to investigate the difference in the underlying architecture of the annotation groups across populations and make the method more robust for transethnic risk prediction. Secondly, although the posterior genetic effect estimates are shrunk towards zero by PRSbils, they are not exactly zero, which can have negative effects on the predictive accuracy. It remains for future work to make the posterior effect estimates sparser by selecting the groups of annotations to be in the final model. Thirdly, when the goal is to estimate the effect of polygenic risk score on quantitative phenotypes, additional information such as treatment effects can be included in the model to make the estimate more accurate. Our study also demonstrated the potential predictive improvement when the similarity information was combined with existing PRS methods, which provided an analytical basis on which further exploration can be made to incorporate additional information into the framework.

## Conclusion

We propose PRSbils, a PRS method which incorporates the functional annotation information and accounts for the annotation group-wise sparsity by applying a bilevel continuous shrinkage prior on the genetic effects. PRSbils uses summary statistics to get the posterior genetic effect estimates for each functional annotation group, estimates the combination weights of group-level PRS using a separate set of individual-level data, and generates the final score. We have shown in this study that leveraging the group-wise sparsity architecture of the genetic effects can help improve the performance of polygenic risk prediction. PRSbils is capable of utilizing a wide range of functional annotations, both overlapping and nonoverlapping, into the analysis and remains computationally efficient compared to the benchmark method. In summary, PRSbils enables the efficient and flexible use of different types of annotation information to improve PRS prediction.

## Data and Code Availability

The code generated during this study are available at https://github.com/styvon/PRSbils

## Figures and Tables

**Table 1.** Summary of parameter settings in the simulation study. A total of *M* = 125,000 SNPs were sampled from the UK Biobank data with quality control filters, with 1KG as LD reference panel. The sampled variants were then assigned to *K* different annotation groups, which explain *q*_1_, …, *q*_*K*_% of the total heritability *h*^2^ respectively. For each annotation group *k*, the proportion of causal variants is denoted as *p*. Overlapping pattern I was used in setting 6, where the intersection over union metric (IOU) was higher among the annotation groups with low heritability contribution. For setting 7, overlapping pattern II was used, where IOU was higher among the annotation groups with high heritability contribution.

Setting	*K*	*M*_*k*_	*P*_*k*_ (%)	*q*_*k*_ (%)	Overlap pattern
1	4	49750, 37500, 25125, 12625	0.5, 1, 1.5, 2	0, 0, 10, 90	Non-overlap
2	4	49750, 37500, 25125, 12625	0.5, 1, 1.5, 2	0, 0, 50, 50	Non-overlap
3	4	49750, 37500, 25125, 12625	0.5, 1, 1.5, 2	10, 20, 30, 40	Non-overlap
4	4	49750, 37500, 25125, 12625	0.5, 1, 1.5, 2	25,25,25,25	Non-overlap
5	10	12375 × 6, 12625, 12625, 12750, 12750	0 × 6, 2, 2, 3, 3	0 × 8,10, 90	Non-overlap
6	4	49750, 37500, 25125, 12625	0.5, 1, 1.5, 2	0, 0, 10, 90	Overlap I
7	4	49750, 37500, 25125, 12625	0.5, 1, 1.5, 2	0, 0, 10, 90	Overlap II
